# Ribose-5-phosphate metabolism protects *E. coli* from antibiotic lethality

**DOI:** 10.1128/mbio.00654-25

**Published:** 2025-07-02

**Authors:** Tatyana Seregina, Rustem Shakulov, Giulio Quarta, Konstantin Shatalin, Svetlana Sklyarova, Irina Petrushanko, Artemy P. Fedulov, Alexander V. Ivanov, Vladimir Mitkevich, Alexander Makarov, Alexander S. Mironov, Evgeny Nudler

**Affiliations:** 1 Department of Molecular Biology, Engelhardt Institute of Molecular Biology, Russian Academy of Science, Moscow, Russia; 2 Department of Biochemistry and Molecular Pharmacology, New York University Grossman School of Medicine, New York, New York, USA; 3 Howard Hughes Medical Institute, New York University Langone Health, New York, New York, USA; Duke University School of Medicine, Durham, North Carolina, USA

**Keywords:** *Escherichia coli*, anabolic pentose phosphate pathway, R5P, oxidative stress, antibiotics

## Abstract

In *Escherichia coli*, ribose-5-phosphate (R5P) biosynthesis occurs via two distinct pathways: an oxidative branch of the pentose phosphate pathway (PPP) originating from glucose-6-phosphate, and a reversed non-oxidative branch originating from fructose-6-phosphate, which relies on transaldolases TalA and TalB. Remarkably, we found that disrupting the oxidative PPP branch by deleting the *zwf* gene significantly increased bacterial susceptibility to killing by a variety of antibiotics. Surprisingly, additional mutations in the *talA* and *talB* genes further enhanced bacterial sensitivity to oxidative stress and antibiotic-mediated killing though they had little impact on the minimal inhibitory concentrations (MICs). The hypersensitivity observed in the *zwf talAB* mutant could be fully reversed by the processes that either utilize R5P or limited its accumulation. Specifically, activating the purine biosynthetic regulon or inhibiting nucleoside catabolism via *deoB* gene inactivation, which blocks the conversion of ribose-1-phosphate to R5P, restored bacterial tolerance. Furthermore, enhancing the biosynthesis of cell wall component ADP-heptose from sedoheptulose-7-phosphate suppressed antibiotic killing of the *zwf talAB* mutant. Biochemical analysis confirmed a direct link between elevated intracellular R5P levels and increased bacterial susceptibility to antibiotics-induced killing. These findings suggest that targeting the PPP could be a promising strategy for developing new therapeutic approaches aimed at potentiating clinically relevant antibiotics.

**IMPORTANCE:**

Recent studies have revealed the crucial role of bacterial cell’s metabolic status in its susceptibility to the lethal action of antibacterial drugs. However, there is still no clear understanding of which key metabolic nodes are optimal targets to improve the effectiveness of bacterial infection treatment. Our study establishes that the disruption of the canonical pentose phosphate pathway induces one-way anabolic synthesis of pentose phosphates (aPPP) in *E. coli* cells, increasing the killing efficiency of various antibiotics. It is also demonstrated that the activation of ribose-5-phosphate utilization processes restores bacterial tolerance to antibiotics. We consider the synthesis of ribose-5-phosphate to be one of the determining factors of bacterial cell stress resistance. Understanding bacterial metabolic pathways, particularly the aPPP’s role in antibiotic sensitivity, offers insights for developing novel adjuvant therapeutic strategies to enhance antibiotic potency.

## INTRODUCTION

It is now recognized that the ability of antibiotics to eradicate bacteria is closely tied to their metabolic state, encompassing biosynthetic rates, respiration, and redox balance (1–5). Nonetheless, the specific metabolic mechanisms influencing antibiotic tolerance remain incompletely understood.

**Fig 1:**
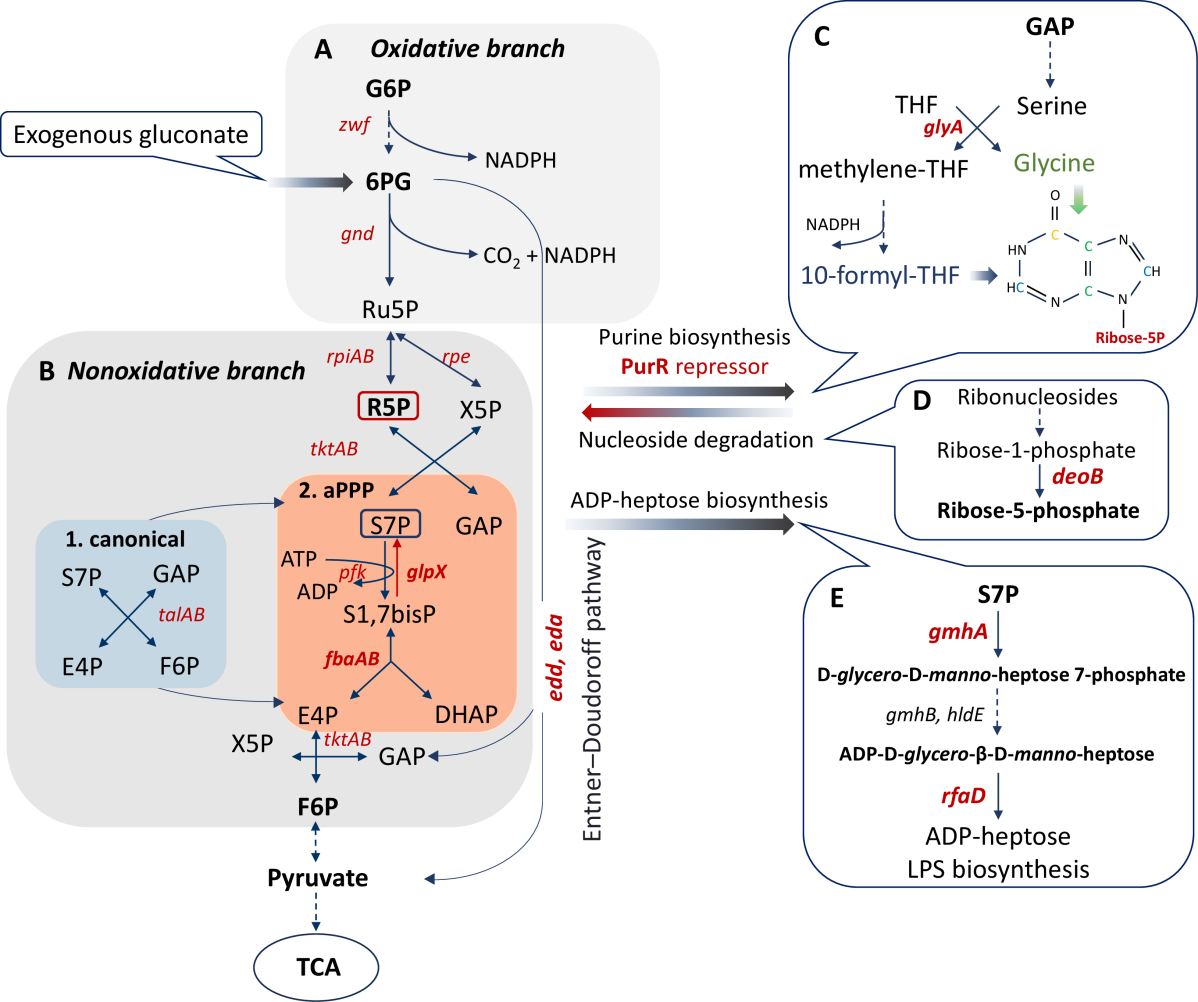
Scheme demonstrating the processes of synthesis and assimilation of R5P in *E. coli* cells. The anabolic synthesis of pentose phosphates (aPPP) occurs in *E. coli* upon inactivation of the oxidative branch by *zwf* mutation (**A**) and replacement activity of transaldolase TalAB (**B1**) by the glycolysis enzymes aldolase FbaA, phosphatase GlpX, and phosphofructokinase Pfk (**B2**). (**C**) Derepression of purine nucleotide synthesis by inactivation of the PurR repressor enhances the efflux of R5P from the PPP. The serine-glycine pathway is also under the control of PurR and is another source of generation of reducing equivalents of NADPH. (**D**) Catabolism of ribonucleosides makes a significant contribution to the intracellular pool of R5P. The final stage of the conversion of ribose-1-phosphate to R5P is carried out by phosphopentomutase *deoB*. (**E**) Sedoheptulose-7-phosphate, which is involved in the formation of the futile cycle of aPPP, serves as a precursor to the lipopolysaride component ADP-heptose. Abbreviations used: G6P, glucose-6-phosphate; 6 PG, 6-phosphogluconate; Ru5P, ribulose-5-phosphate; R5P, ribose-5-phosphate; X5P, xylulose-5-phosphate; S7P, sedoheptulose-7-phosphate; DHAP, dihydroxyacetone phosphate; E4P, erythrose-4-phosphate; F6P, fructose-6-phosphate; GAP, glyceraldehyde-3-phosphate; THF, tetrahydrofolate; TCA, tricarboxylic acid cycle.

The pentose phosphate pathway (PPP) serves as a pivotal metabolic hub for pentose phosphate synthesis. The canonical PPP comprises two branches: oxidative decarboxylation of glucose-derived phosphogluconate, generating NADPH for anabolic processes and oxidative stress defense, and the non-oxidative branch, which lacks a driving force-generating reaction for pentose phosphate production (6). Certain organisms, such as *Prevotella copri*, utilize modified non-oxidative PPP variants to assimilate carbon sources like xylose (7–13). This includes anabolic aPPP, where fructose-6-phosphate and glyceraldehyde-3-phosphate are converted to sedoheptulose-1,7-bisphosphate and sedoheptulose-7-phosphate using aldolase A and phosphatase GlpX enzymes, bypassing transaldolase (Fig. 1) (7, 14).

Here, we demonstrate that disrupting the canonical PPP in a triple *zwf talAB* mutant, leading to aPPP, heightens the lethal action of diverse bactericidal antibiotics on *E. coli*. We propose that the distinctive R5P metabolism in the *zwf talAB* mutant underlies the increased killing effect of antibiotics. This suggestion is supported by the identification of mutations and factors mitigating the hyper-susceptibility of the *zwf talAB* mutant.

## RESULTS

### Disruption of the canonical PPP increases antibiotic killing efficiency

Inactivating the oxidative branch of the PPP by deleting the *zwf* gene, which encodes glucose 6-phosphate dehydrogenase, did not affect *E. coli* growth (Fig. S1). This resilience is due to pentose phosphate synthesis from fructose-6-phosphate and glyceraldehyde-3-phosphate through the non-oxidative PPP branch, utilizing transketolases and transaldolases (15). Cells lacking transaldolases (*∆talAB*) remained viable (Fig. S1), likely due to compensatory activities from aldolase and 6-phosphofructokinase I (*pfk*) (10), along with the activity of fructose-1,6-phosphatase, acting as sedoheptulose-1,7-phosphatase encoded by *glpX* gene (16, 17). Indeed, the triple *∆zwf ∆talAB* mutant exhibits increased aldolase activity (Fig. S2A) and elevated *glpX* expression (Fig. S2B). Accordingly, *glpX* inactivation in the *∆zwf ∆talAB* background resulted in a failure to form colonies (Fig. S3). Consequently, cells deficient in the oxidative branch of PPP spontaneously shifted to the anabolic PPP (aPPP), generating pentose phosphates through the intermediate metabolite sedoheptulose-1,7-phosphate (Fig. 1B).

**Fig 2:**
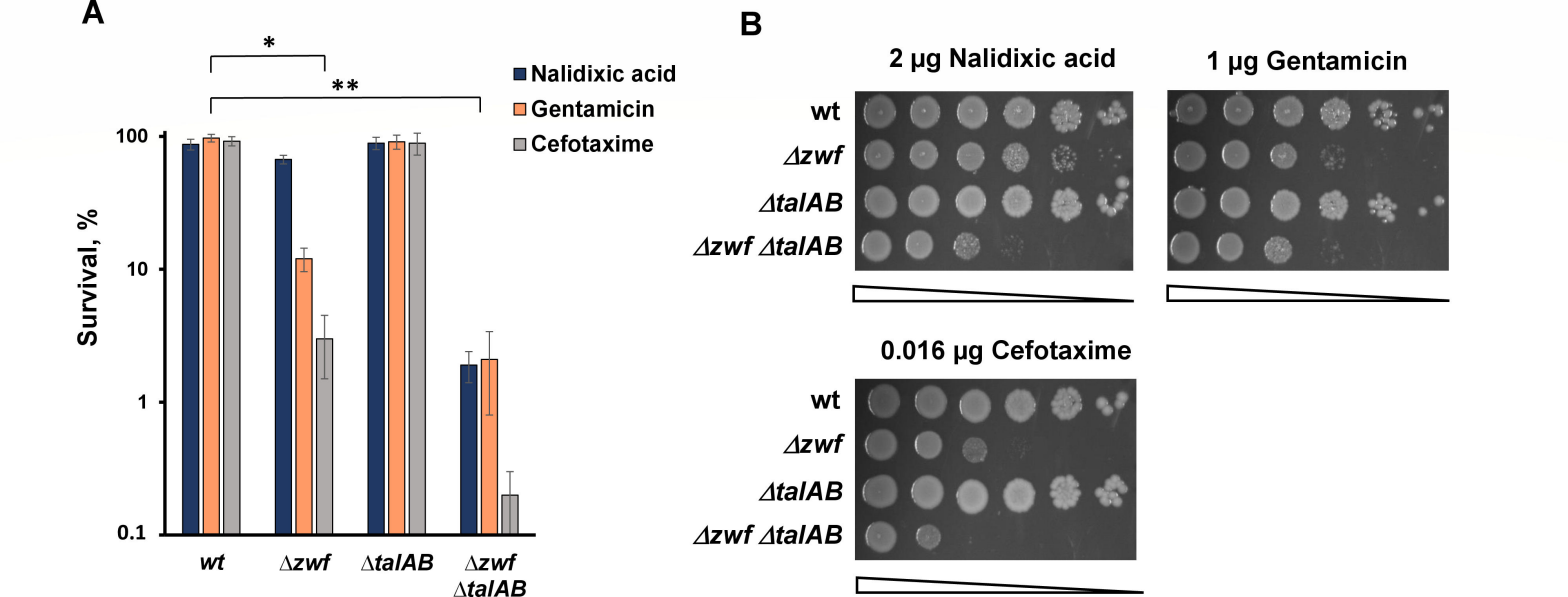
Sensitivity of *E. coli zwf talAB* mutants to different classes of antibiotics. (**A**) Overnight cultures of indicated *E. coli* strains were diluted with fresh LB 1:100 and grown to ∼2 × 10^7^. Suspensions of *E. coli* cells treated with the antibiotics nalidixic acid, gentamicin, and cefotaxime, at 5 × MIC for 1 h. Cell survival was determined by counting cfu and is shown as the mean ± SD from three independent experiments. *P < 0.05, compared to the wild-type cells. **P < 0.01, compared to the wild-type cells. (**B**) Representative efficiencies of colony formation of WT (MG1655) and mutant *E. coli* cells in the presence of various antibiotics (2 µg nalidixic acid, 1 µg gentamicin, 16 ng cefotaxime). Cells were spotted on LB agar plates in serial 10-fold dilutions and incubated at 37°C for 24 h.

**Fig 3:**
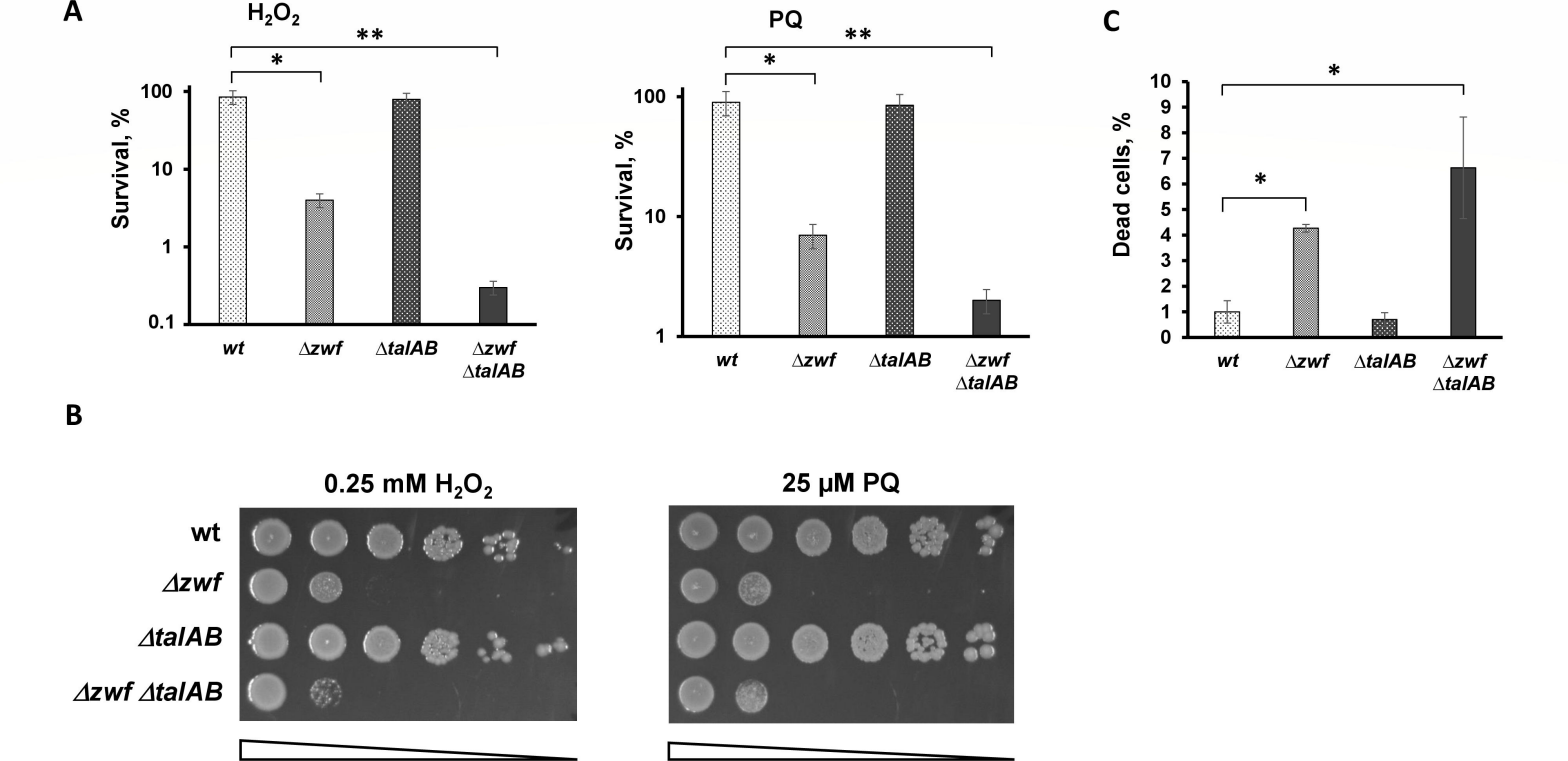
Deletion of *zwf talAB* sensitizes cells to oxidative stress and decreases cell’s viability. (**A**) Overnight cultures of indicated *E. coli* strains were diluted with fresh LB 1:100 and grown to ∼2 × 10^7^. H_2_O_2_ was added to 1.5 mM or 100 µM paraquat for 10 min. Cell survival was determined by counting cfu and is shown as the mean ± SD from three independent experiments. *P < 0.05, compared to the wild-type cells. **P < 0.01, compared to the wild-type cells. (**B**) Representative efficiencies of colony formation of WT (MG1655) and mutant *E. coli* cells in the presence of 0.25 mM H_2_O_2_ or 25 µM paraquat. Cells were spotted on LB agar plates in serial 10-fold dilutions and incubated at 37°C for 24 h. (**C**) The number of dead cells in the cell population was determined by flow cytometry using propidium iodide. Cells were grown in an LB medium to an optical density of 0.5 and then washed twice with phosphate-buffered saline (1×PBS), centrifuged, the supernatant was removed, and cells were resuspended in 100 µL of PBS. Propidium iodide was added at a concentration of 10 µg/mL per min before the start of the analysis. Mean values ± SD from at least three independent experiments are shown. *P < 0.05, compared to the wild-type cells.

Remarkably, unlike the *∆talAB* mutant, the single *∆zwf* mutant exhibited sensitivity to a variety of antibiotics, including quinolones, beta-lactams, and aminoglycosides, as indicated by colony-forming unit (CFU) counts on drug-free medium (Fig. 2A), with minimal impact on the minimal inhibitory concentration (MIC) (Table S1). Unexpectedly, antibiotic sensitivity increased even further when both branches of the PPP were inactivated in the *∆zwf ∆talAB* double mutant (Fig. 2A and B). Introduction of a plasmid carrying the wild-type *zwf* allele completely suppresses the antibiotic sensitivity of the triple mutant ∆*zwf* ∆*talAB* (Fig. S4). Moreover, *zwf*- and *talAB*-deficient strains were hyper-susceptible to exogenous oxidants, such as hydrogen peroxide and paraquat, compared to wild-type (WT) cells (Fig. 3A and B). Notably, both the *∆zwf* and *∆zwf ∆talAB* mutants displayed similar sensitivity to paraquat, but the *∆zwf ∆talAB* mutant showed approximately a one-log increase in H_2_O_2_-induced killing compared to the *∆zwf* mutant (Fig. 3A and B). The *∆zwf* and *∆zwf ∆talAB* mutants also demonstrated a significant increase in spontaneous cell death during exponential growth (Fig. 3C) though no differences in cell death were observed in overnight cultures (Fig. S5).

### Disruption of the canonical PPP leads to cellular redox imbalance

**Fig 4:**
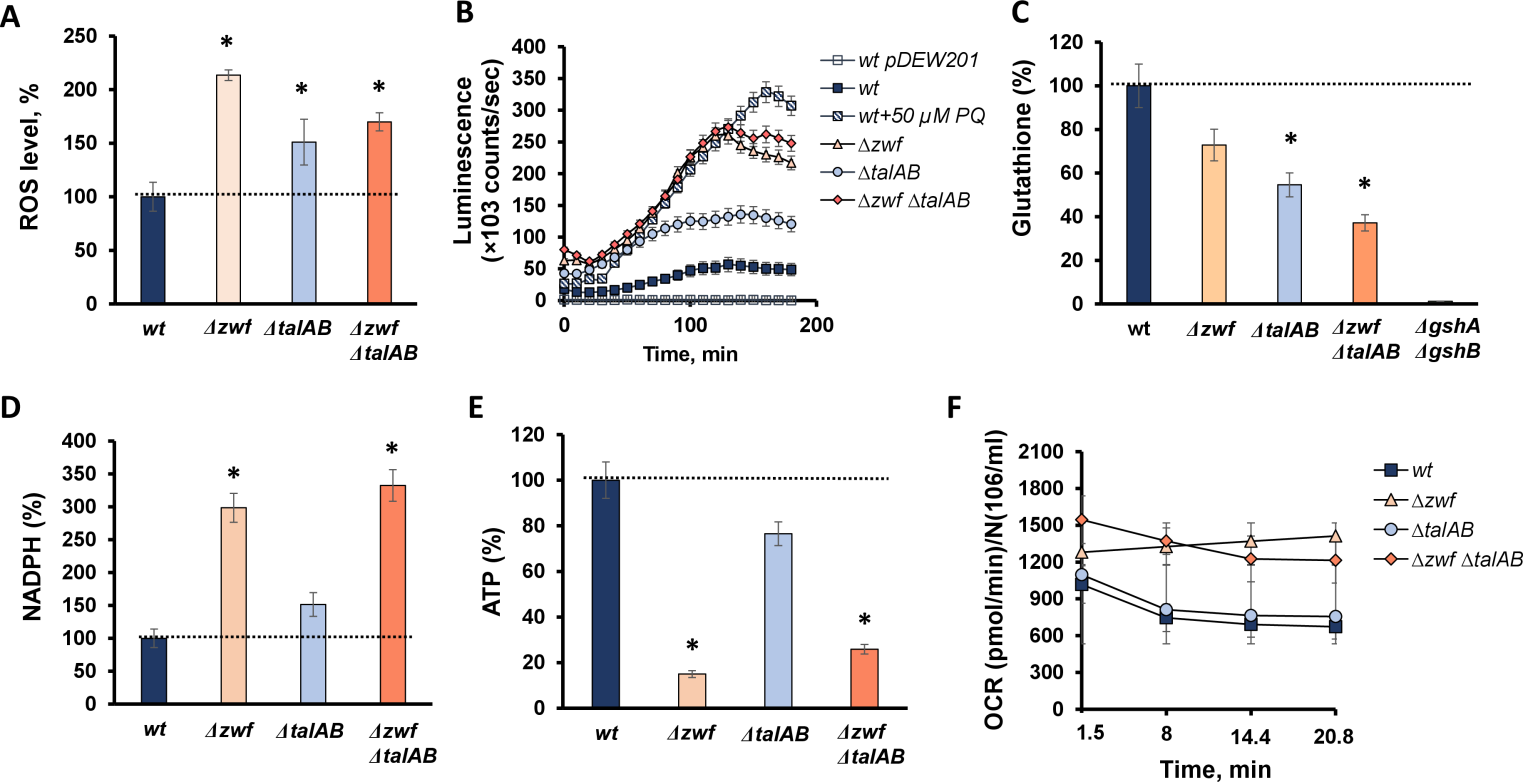
Phenotypic characteristics of ∆*zwf* ∆*talAB* mutants: ROS formation (**A**), activity of the soxS promoter (*wt pDEW201* is a negative control without *PsoxS*, *wt* with added paraquat is a positive control) (**B**), glutathione (GSH) levels (*ΔgshA ΔgshB* with inactivated glutathione synthesis is a negative control) (**C**), content of NADPH (**D**), ATP (**E**), and basal oxygen consumption rate (**F**). Mean values ± SD from at least three independent experiments are shown. *P < 0.05, compared to the wild-type cells.

Oxidative stress is known to contribute to antibiotic toxicity (3, 18, 19). To better understand the hypersensitivity of aPPP cells to antibiotics, we examined the redox status in individual *∆zwf*, *∆talAB*, and triple *∆zwf ∆talAB* mutants. All mutants showed a significant accumulation of endogenous reactive oxygen species (ROS) compared to WT cells, as indicated by staining with the ROS-specific dye dihydrorhodamine 123 (Fig. 4A). This observation was further supported by the activation of the oxidative stress-inducible *soxS* promoter (Fig. 4B). Also, all mutants displayed reduced levels of glutathione, a major endogenous antioxidant, with the most substantial decrease observed in the triple *∆zwf ∆talAB* mutant (Fig. 4C). Another notable redox-related feature, observed in all mutants except the double *talAB* mutant, was elevated levels of NADPH (Fig. 4D), indicating a global redox imbalance following inactivation of the oxidative branch in *∆zwf* and *∆zwf ∆talAB* cells.

We hypothesized that in the absence of PPP, a compensatory NADPH-generating energy-consuming process could sustain high levels of NADPH, potentially explaining the observed drop in ATP content (Fig. 4E). Indeed, the hyperactivity of the serine-glycine pathway associated with purine biosynthesis may account for the increased NADPH levels in *zwf* mutant, as demonstrated by their return to normal levels upon inactivation of serine hydroxymethyltransferase (*glyA*) in ∆*zwf* cells (Fig. S2C) (20). Importantly, the deletion of *glyA* in the WT parent strain did not significantly alter NADPH levels (Fig. S2C).

### R5P overflow underlies antibiotic hypersensitivity

Under antibiotic exposure, *∆zwf ∆talAB* cells are expected to accumulate R5P due to the unidirectional nature of the anabolic pentose phosphate pathway (aPPP) process (Fig. 1B2). We hypothesize that the hypersensitivity of aPPP cells to antibiotics and the observed changes in redox status may arise from an imbalance between R5P synthesis and its metabolic consumption. Processes that increase anabolic activity involving R5P, as well as those that decrease R5P biosynthesis, are anticipated to alleviate the *∆zwf ∆talAB* phenotypes. We ruled out the influence of exogenous ribose transport from our analysis, as inactivation of the ribose transporter gene *rbsD* and the addition of exogenous ribose to LB medium do not affect the antibiotic sensitivity of the triple mutant (Fig. S6).

**Fig 5:**
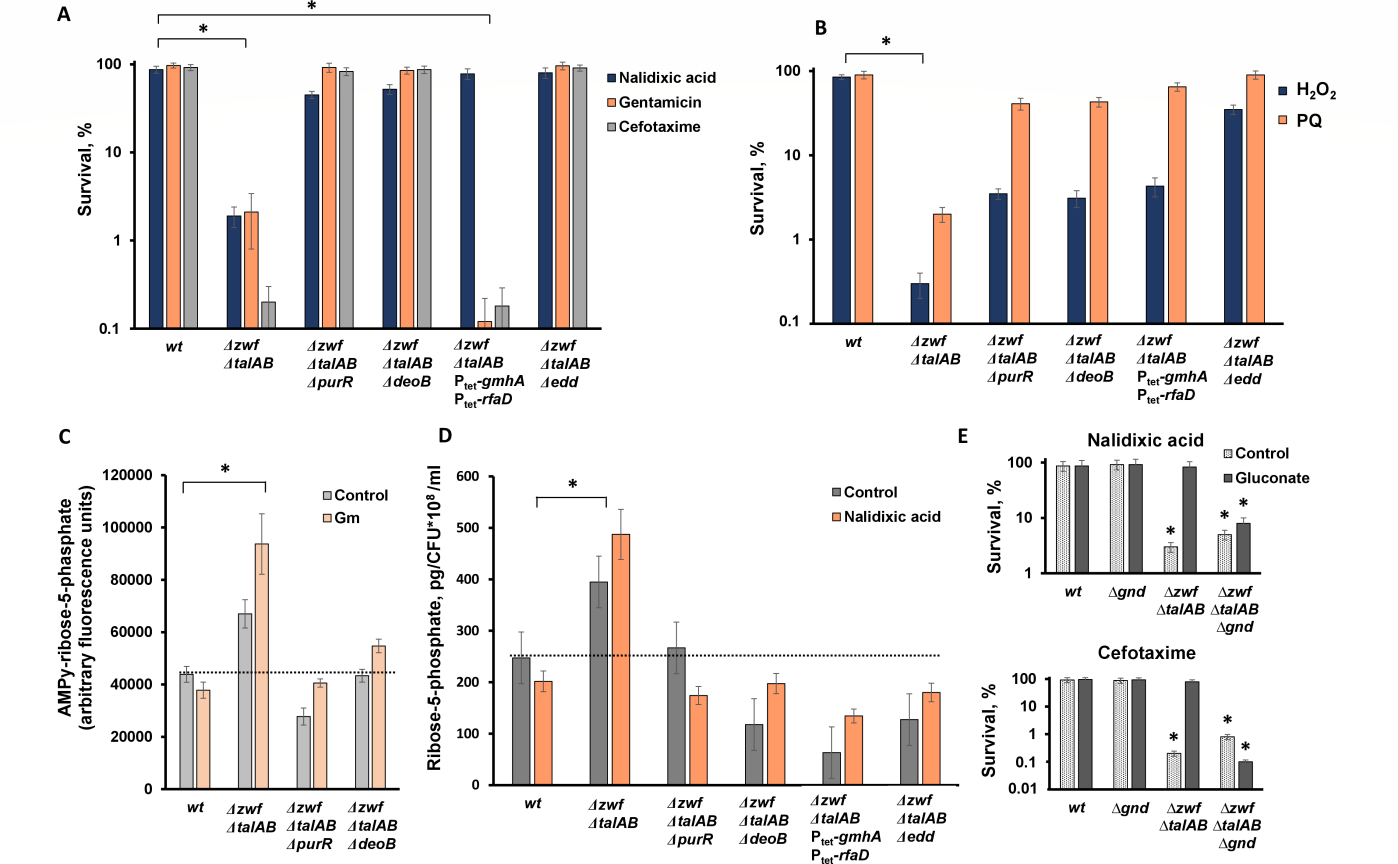
Suppression of aPPP phenotypes and correlation with the R5P pool. Hypersensitivity to antibiotics (**A**) and redox imbalance (**B**) can be suppressed by stimulating R5P utilization via enhanced purine metabolism (∆*purR*) or R5P incorporation into the cell wall (Ptet-*gmhA* Ptet-*rfaD*), or by limiting the formation of R5P from nucleosides (*deoB*). Reactivation of the PPP via blocking the Entner-Doudoroff pathway (Δ*edd*) completely suppresses the antibiotic sensitivity of the *zwf talAB* mutant. (**C**) R5P concentrations from HPLC analysis: Overnight cultures were grown to mid-log phase, and metabolites were extracted and fluorescently labeled (see Materials and Methods). Metabolites were extracted in triplicate and analyzed by HPLC. The average R5P concentration from each strain is shown. Mean values ± SD from at least three independent experiments are presented. *P < 0.05, compared to wild-type cells. (**D**) R5P quantification by GC-MS: R5P was quantified by gas chromatography-mass spectrometry (GC-MS) using overnight cultures grown to mid-log phase (see Materials and Methods). Mean values ± SD from at least three independent experiments are shown. *P < 0.05, compared to wild-type cells. (**E**) The addition of sodium gluconate to the growth medium restores the antibiotic tolerance of the ∆*zwf* ∆*talAB* mutant to the level of wild-type cells. Deletion of the *gnd* gene removes the suppressive effect of sodium gluconate. Overnight cultures of the indicated *E. coli* strains were diluted 1:100 into fresh LB and grown to ∼2 × 10^7^ cells. Suspensions of *E. coli* cells were treated with antibiotics at 5 × MIC for 1 h or with H_2_O_2_ or paraquat for 10 min. Cell survival was determined by counting CFUs, and the results are shown as the mean ± SD from three independent experiments. *P < 0.05, compared to wild-type cells.

A primary metabolic process utilizing R5P is nucleotide biosynthesis. Remarkably, the derepression of the purine biosynthetic regulon via PurR repressor inactivation enhances the tolerance of *∆zwf ∆talAB* cells to antibiotics (Fig. 5A) and oxidants (Fig. 5B).

Furthermore, activation of cell wall biosynthesis, which consumes a significant portion of cellular pentose phosphates, also mitigates the hypersensitivity of *∆zwf ∆talAB* to antibiotics and oxidants (Fig. 5A and B; Fig. S7). The initial step in ADP-heptose formation for lipopolysaccharide (LPS) envelope biogenesis is catalyzed by D-sedoheptulose 7-phosphate isomerase (*gmhA*), followed by subsequent steps involving ADP-L-glycero-D-mannoheptose 6-epimerase (*rfaD*) (21). Overexpression of *gmhA* and *rfaD* genes increases R5P flux into sedoheptulose and subsequently into cell wall LPS (22). Consequently, placing chromosomal copies of *gmhA* and *rfaD* under the control of the strong constitutive P_tet_ promoter effectively suppresses ∆*zwf* ∆*talAB* cells’ hypersensitivity to nalidixic acid and oxidants (Fig. 5A and B; Fig. S7). However, the opposite effect is observed with respect to cefotaxime and gentamicin (Fig. 5A): overexpression of the *rfaD* gene increases the sensitivity of the parental strain to these antibiotics (Fig. S8).

In rich LB medium, the *deo* operon, responsible for purine and pyrimidine nucleoside catabolism, significantly contributes to the R5P pool (17, 23). Thus, reducing nucleoside catabolism was expected to alleviate aPPP cells’ sensitivity to antibiotics. The final step in nucleoside degradation is the conversion of ribose-1-phosphate to R5P, catalyzed by phosphopentomutase (*deoB*). As anticipated, *deoB* gene deletion strongly mitigates *∆zwf ∆talAB* cells’ hypersensitivity to oxidative stress and antibiotics (Fig. 5A and B; Fig. S7).

To directly test the role of R5P in antibiotic tolerance, we measured intracellular R5P levels in mutant cells (Fig. 5C and D ). As expected, the triple mutant *∆zwf ∆talAB* exhibited more than a twofold increase in the R5P pool compared to the WT strain. Notably, the deletion of either *purR* or *deoB* in the *∆zwf ∆talAB* background reduced R5P levels to approximately those of WT cells (Fig. 5C and D).

Therefore, strategies aimed at R5P accumulation, such as inhibiting its utilization or promoting its formation from nucleosides, hold promise for enhancing redox imbalance and reducing antibiotic tolerance.

### Gluconate suppresses effect of *zwf* deletion and restores canonical PPP

The deletion of the *zwf* gene prevents ribose synthesis from glucose-6-phosphate via the oxidative branch of the pentose phosphate pathway (PPP). However, the downstream part of this pathway, involving the decarboxylation of 6-phosphogluconate to form ribulose-5-phosphate and reduced NADP^+^, remains intact. We hypothesized that excessive exogenous gluconate would “reactivate” the PPP in ∆*zwf* ∆*talAB* cells by promoting its final step in the oxidative branch. When gluconate is added to LB medium, it is phosphorylated to gluconate-6P, which undergoes decarboxylation to form CO2 and NADPH, subsequently converting to ribulose-5P and then to ribose-5P. Additionally, gluconate-6P can be metabolized through the Entner-Doudoroff pathway into lower glycolysis products, glyceraldehyde-3P and pyruvate. Thus, the addition of gluconate to the medium may mimic the activity of the intact pentose phosphate pathway in the ∆*zwf* ∆*talAB* triple mutant, coupling ribose-5P and NADPH biosynthesis despite the absence of Zwf activity. As anticipated, the addition of sodium gluconate to the growth medium restored antibiotic tolerance in the ∆*zwf* ∆*talAB* mutant to levels comparable to the wild type (Fig. 5E). This suppressive effect of sodium gluconate was completely abolished by the deletion of the *gnd* gene encoding phosphogluconate dehydrogenase, the enzyme responsible for the final step of the oxidative branch of PPP (Fig. 5E).

Excess phosphogluconate in the cell can also be achieved by blocking the Entner-Doudoroff pathway (24), where the initial step is catalyzed by phosphogluconate dehydratase (*edd*). Inactivation of the *edd* gene in the *∆zwf ∆talAB* strain completely suppressed the sensitivity of *∆zwf ∆talAB* cells to all antibiotics used in our study (Fig. 5A; Fig. S7). The suppression of antibiotic sensitivity in the ∆*zwf* ∆*talAB* triple mutant, in the context of *edd* deletion, is associated with a decrease in the intracellular R5P pool (Fig. 5D). These results suggest that gluconate metabolism overcame the anabolic PPP in PPP-deficient *∆zwf ∆talAB* cells, effectively mimicking normal PPP functioning, in which ribose-5-phosphate formation is coupled with NADP^+^ reduction.

Remarkably, all types of suppressions, described above of hypersensitivity of *∆zwf ∆talAB* cells to antibiotics (*purR*, *deoB*, Ptet-*lpcA* Ptet-*rfaD*, *edd* mutations and gluconate addition), consistently accompanied the restoration of intracellular glutathione content (Fig. S9A) and ATP levels (Fig. S9B), nearly to the level of WT cells. Moreover, the suppression of antibiotic hypersensitivity was associated with a reduction in the level of reduced NADPH (Fig. S9C), suggesting an inverse correlation between intracellular glutathione and NADPH levels. The suppressive effect associated with *purR*, *deoB*, and *edd* deletions can be reversed by introducing wild-type alleles of the corresponding genes (Fig. S4).

## DISCUSSION

It has been observed that when both branches of the canonical pentose phosphate pathway (PPP) are shut down, microorganisms can switch to alternative anabolic synthesis of pentose phosphates (13–15). Our findings indicate that the appearance of aPPP in *E. coli* changes redox status, decreases viability, and enhances sensitivity to a wide range of classes of bactericidal antibiotics with different primary targets. These results align with the findings of Wang et al. (25), who reported heightened sensitivity of *talA* and *zwf* mutants to gentamicin. Furthermore, aPPP causes significant redox imbalance, characterized by elevated endogenous ROS and NADPH levels and decreased levels of glutathione and ATP (Fig. 4). The activation of aPPP also leads to increased respiration, indicating intensified metabolic activity (Fig. 4F). The deletion of *zwf*, which is part of the redox-sensing SoxRS regulon, appears to decouple the oxidative stress response from the coordinated generation of reducing equivalents (NADPH) and pentose phosphates biosynthesis.

We obtained direct evidence that enhanced sensitivity of the triple mutant *∆zwf ∆talAB* to various classes of antibiotics correlates with an increased intracellular R5P pool (Fig. 5). Recent studies have highlighted the important regulatory role of R5P plays in coordinating nucleotide recycling and amino acid synthesis in the stringent response (26–28). Furthermore, in our previous study, we found that impaired efflux of the sedoheptulose-7-phosphate (via *gmhA* deletion) from the PPP leads to a significant increase in bacteria sensitivity to antibiotics, which can be suppressed by activating purine biosynthesis through *purR* deletion (29). Strengthening the processes of pentose phosphate consumption by promoting purine biosynthesis via *purR*, or stimulating cell envelope synthesis through Ptet-*gmhA* and Ptet-*rfaD*, or limiting R5P formation as a result of nucleoside degradation through *deoB*, leads to the restoration of antibiotic tolerance except cefotaxime and gentamicin and specific physiological parameters in aPPP cells (Fig. 5; Fig. S7 and S9). We consider that uncoupling the synthesis of R5P with NADP^+^ reduction to be one of the crucial factors of bacterial cell stress tolerance.

In summary, the findings presented in this study suggest that the induction of unidirectional aPPP is a critical factor in the ability of bacteria to tolerate antibiotics. Therefore, manipulating the two ways of pentose phosphate generation (oxidative branch of canonical PPP and aPPP pathway) may have therapeutic potential to enhance antibiotic efficacy and combat antibiotic resistance.

## MATERIALS AND METHODS

### Strains and growth conditions

All *E. coli* strains utilized in this work are listed in Table S2. BW25113 and its derivatives (single gene deletion mutants) were obtained from the *E. coli* Keio Knockout Collection (Thermo Scientific) (30). P1 transduction was employed to introduce mutations into new strains (31). When necessary, Cam or Kan drug resistance markers were excised from strains using the FLP activity of pCP20, followed by loss of the plasmid at the nonpermissive temperature (32). All mutations were verified by PCR and gel analysis. DNA manipulation and the transformation of *E. coli* strains were performed according to standard methods (33). LB complete medium was used for the routine growth of *E. coli*. When appropriate, antibiotics were added at 40 µg/mL (for kanamycin), 30 µg/mL (for chloramphenicol), and 100 µg/mL (for ampicillin). For solid medium, 1.5% agar was added.

### P_tet_-*gmhA* and P_tet_-*rfaD* constructions

To construct the *gmhA* and *rfaD* overexpression strains, the native promoter of these genes was substituted by PLtet-O1 (34). Briefly, the P Ltet-O1-attL-CmR-attR cassette integrated into the AM3009 strain *mstA* gene (19) was amplified with primers 5′-gcacttcaggtcaaaaagtcctggtcatagcacctgcgctcaagttagtataaaaaagct-3′ and 5′-ttcgttcag-ttcgttacgaataagatcctggtacatggtacctttctcctctttaatga-3′ (for *gmhA* gene) and 5′- ttcacatgcaaaaccaacatccgccatgaaggactacgctcaagttagtataaaaaagct-3′ and 5′-gccgat-aaagcccgcgccgccggtaacgatgatcatggtacctttctcctctttaatga-3′ (for *rfaD* gene). The first set of primers contained the upstream region of *gmhA* and *rfaD* genes and the sequence of attR, while the second set of primers contained the coding region of corresponding genes and the sequence of PLtet-O1. The PCR fragments were transformed into MG1655 containing pKD46 (32). CmR clones were tested in the presence of the PLtet-O1-attL-CmR-attR cassette by PCR with primers 5′-aacaaagctcacattgttgct-3′ and 5′-gcgctgaatggcgtgaatatt-3′ (for *gmhA* gene) and 5′-atcggaatattgatactaaagc-3′ and 5′-aatatcggtgatgcctttatc-3′ (for *rfaD* gene). All constructs were sequenced for verification and introduced into corresponding chromosomal loci according to ref (31). All strains bearing Ptet constructs do not contain the *tetR* gene and, therefore, exhibit constitutive expression of target genes.

### Determination of sensitivity to antibiotics

Overnight bacterial cultures were diluted 100 times with fresh LB medium and grown to OD_600nm_ ≈ 0.5 under aerobic conditions at 37°C. Cell suspensions were aligned in optical density, and a series of 10-fold dilutions were prepared in a 96-well plate. Ready dilutions were plated on plates with a rich agar medium containing various concentrations of the studied antibiotics: nalidixic acid, cefotaxime, gentamicin, erythromycin, and rifampicin as described in reference 35. The dishes were incubated overnight at 37°C, and the result was photographed on a GelCamera M-26XV detection system. For antibiotic survival assays, overnight bacterial cultures were diluted 100-fold and grown at 37°C to ∼2 × 10^7^ cells per mL, treated with the indicated concentration of nalidixic acid, cefotaxime, or gentamicin, and after 60 min of incubation, samples were diluted and plated on LB agar and incubated at 37°C for 24 h. Cell survival was determined by counting cfu and is shown as the mean ± SD from three independent experiments.

Standardized MICs were determined by the modified broth microdilution method specified by the Clinical and Laboratory Standards Institute (35). Briefly, the test antibiotic was serially diluted twofold in 100 µL LB. The bacteria inoculum was 100 µL of a 1.0 × 10^6^ cfu/mL dilution in LB. The MIC was the lowest concentration of antibiotic that prevented turbidity after 24 h of incubation at 37°C. The mean value ± SD from three independent experiments.

### Genetic complementation of deletions of *zwf*, *purR*, *deoB*, and *edd*

A complementation test was performed to confirm the association of the observed antibiotic sensitivity phenotypes in the triple mutant *∆zwf ∆talAB* and the *∆zwf ∆talAB* mutants carrying suppressive deletions (*purR*, *deoB*, and *edd*). Plasmids carrying the wild-type alleles of the *zwf*, *purR*, *deoB*, and *edd* genes were used for complementation. The structural regions of these genes were cloned into the pUC19 vector at the BamHI and EcoRI sites using primers listed in Table S3. All constructs were verified by sequencing. The resulting plasmids were transformed into the strains ∆*zwf ∆talAB, ∆zwf ∆talAB ∆purR, ∆zwf ∆talAB ∆deoB, and ∆zwf ∆talAB ∆edd,* and antibiotic susceptibility was tested as described above.

### Detection of pSoxS activity

The pSoxS::lux plasmid (lux biosensor) containing the *Photorhabdus luminescens* lux operon genes under the control of the *soxS* gene promoter was used as a reporter system for detecting the formation of reactive oxygen species (36). All studied strains were transformed with this plasmid. Strain MG1655, transformed with the plasmid pDEW201 containing the *luxCDABE* operon without a promoter, was used as a negative control (37). Overnight cultures of the strains were diluted 100 times and grown to OD_600nm_ ≈ 0.2. Suspensions were normalized by optical density and transferred to a 96-well plate in 200 µL. The detection of bioluminescence was carried out in a tablet reader Tecan Spark at a wavelength of 490 nm for 3 h at room temperature.

### Determination of viability of cells and ROS using flow cytometry

Cells were grown in complete medium to an optical density of 0.5 and then washed twice with phosphate-buffered saline (1×PBS), centrifuged, the supernatant was removed, and cells were resuspended in 100 µL of PBS. Cell parameters were analyzed using flow cytometry on a BD LCR Fortessa flow cytometer (Becton Dickinson, Franklin Lakes, New Jersey, USA). The cell population for analysis was selected according to the parameters of forward (FSC) and side scattering (SSC), which characterize the size and granularity of cells. The percentage of dead cells in the cell population was assessed using propidium iodide (Ex/Em = 535/617 nm, Sigma-Aldrich, St. Louis, Missouri, USA), which was added to the cells at a concentration of 10 µg/mL per min before the start of the analysis. Propidium iodide penetrates the cells with a damaged membrane and, after binding to DNA, has a bright fluorescence in the red region of the spectrum. The ROS level was assessed using the dye Dihydrorhodamine 123 (DHR123) (Ex/Em = 507/525 nm, ThermoFisher Scientific, Waltham, Massachusetts, USA) (38), which was added to the cells to a final concentration of 7.5 µM, and the cells were incubated for an hour in the dark at 37°C.

### Measurement of NADPH level

Measurement of NADPH levels was performed using a fluorimetric NADP/NADPH Assay Kit (Abcam). Cells were grown to OD_600nm_ ≈ 0.5 in a thermostated shaker at 37°C. Preparation of cell extracts, as well as all subsequent manipulations, was carried out according to the manufacturer’s instructions attached to the kit. Sample fluorescence was detected in a Tecan Spark plate reader at Ex/Em = 540/590 nm. The results obtained were related to the optical density of the OD_600nm_ culture.

### Measurement of intracellular glutathione levels

Quantitative determination of the level of intracellular glutathione was carried out by the modified Tietz method (39). One hundred microliters of overnight culture was transferred to 10 mL of fresh LB medium and grown to OD_600nm_ ≈ 0.5. Cells from a 5 mL suspension were pelleted by centrifugation and suspended in a lysis buffer: 0.1 M potassium phosphate buffer with 5 mM EDTA disodium salt, pH 7.5, supplemented with 0.1% Triton X100. The cells were homogenized with a pestle and centrifuged at 11,000 rpm for 5 min at 4°C. Supernatant was transferred into clean test tubes. The reaction mixture of 60 µL of cell extract, 120 µL of DTNB (0.3 mg/mL) and GOR (1.5 units/mL), and 60 µL of NADPH (0.6 mg/mL) was incubated for 3 min at room temperature, after which adsorption was measured at 412 nm. GSH (Sigma Aldrich, USA) was used to construct a calibration curve. The obtained values were referred to the optical density of the cultures.

### ATP level measurement

The intracellular ATP level was determined by the luminescence method using the Adenosine 5′-triphosphate (ATP) Bioluminescent Assay Kit (Sigma Aldrich, USA). Five milliliters of cell suspension grown to OD_600nm_ ≈ 0.5 was rapidly washed twice with saline and suspended in 2 mL of saline. Preparation of the reaction mixture and measurement of luminescence were carried out as described in the manufacturer’s instructions. The obtained values were related to the optical density of the culture and expressed as a percentage. The level of ATP in the wild-type strain was taken as 100%.

### Bacterial respiration

Seahorse XFe24 Analyzer was used to quantitate oxygen consumption rates (OCRs) (5). An overnight of *E. coli* cells was diluted 1:100 into fresh LB media and grown to an OD_600nm_ of ≈ 0.5. Cells were diluted to 5 × the final OD, and 500 µL of diluted cells was added to XF Cell Culture Microplates precoated with poly-D-lysine (PDL) (5). Cells were centrifuged for 10 min at 1,400 × *g* to attach them to the precoated plates. Basal OCR was measured for four cycles (20 min). OCR was normalized to the number of viable cells quantitated using flow cytometry on a BD LCR Fortessa flow cytometer (Becton Dickinson, Franklin Lakes, New Jersey, USA).

### RNA extraction and qRT-PCR

*E. coli* K-12 MG1655, TS011, TS013, and TS015 cells were grown until OD_600nm_ ≈ 0.5, and total RNA was extracted using the RNeasy Mini Kit (QIAGEN) according to the manufacturer’s protocol according to reference 19. All RNA samples were treated with DNaseI (Fermentas). Five hundred nanograms of total RNA were reverse-transcribed with 100U of SuperScript III enzyme from the First-Strand Synthesis Kit for RT-PCR (Invitrogen) according to the manufacturer’s protocol in the presence of appropriate gene-specific primers. One microliter of the reverse transcription reactions was used as a template for real-time PCR. The gene *def* encoding peptide deformylase was used for normalization. Each real-time PCR mixture (25 µL) contained 10 µL SYBR Green I PCR Master Mix (Syntol), 12 µL nuclease-free H_2_O, 1 µL of 10 µM forward primer, 1 µL of 10 µM reverse primer, and 1 µL of cDNA template. Amplifications were carried out using DTlite S1 CyclerSystem (DNA Technology). Reaction products were analyzed using 2% agarose electrophoresis to confirm that the detected signals originated from products of expected lengths. Each qRT-PCR reaction was performed at least in triplicate, and average data are reported. Error bars correspond to the standard deviation.

### Determination of aldolase activity

Aldolase activity was measured using the Aldolase Activity Assay Kit (Colorimetric) (Abcam). Cells were washed with cold saline and concentrated in 100 µL of Aldolase Assay Buffer to a density of 1 × 10^6^ per mL. Further manipulations were performed in accordance with the manufacturer’s recommendations. The obtained values of aldolase activity were related to the concentration of total protein in the studied cell extracts.

### Determination of ribose-5-phosphate

#### HPLC analysis

Overnight cultures were grown in Luria Bertani broth (LB, Difco) at 37°C with shaking. Overnight cultures were diluted 1:1,000 and grown to mid-log OD_600nm_ ≈ 0.5 at 37°C with vigorous shaking in erlenmeyer flasks before 3 × 5 mL samples were collected. Five milliliters of culture was immediately chilled in an ice-water bath for 10 min followed by centrifugation at 5,000 × *g* for 5 min at 4°C and media removed. The cell pellet was then resuspended in 1,000 µL of pre-chilled MeCN:MeOH:Water (40:40:20 vol/vol/vol) and extracted at −20°C for approximately 1 h until all cell lines were processed. The extract was centrifuged at 17,000 × *g* for 20 min at 4°C. Supernatant was transferred to a fresh tube and dried in a vacuum centrifuge at room temperature. To the remainder of cells, gentamicin sulfate was added to 5 µg/mL final and incubated at 37°C with shaking for 60 min. The extraction was then repeated. The dried extracts were dissolved in 100 µL of HPLC grade water at 25°C with vigorous shaking for 10 min followed by brief centrifugation. Derivatization solution was freshly prepared in a chemical hood, consisting of 80 mM *N*-ethyl-*N*′-(3-dimethylaminopropyl) carbodiimide hydrochloride (EDC, Sigma E6383), 400 mM 3-picoylamine (AMPy, Sigma A65409), and 500 mM 4-Methylmorpholine (NMM, Sigma M56557) in a solvent system of 1:1 H_2_O:DMSO (40). As a standard, 100 mM solution of ribose 5-phosphate (Sigma R7750) was prepared simultaneously in water and modified according to the same protocol. One hundred microliters of derivatization solution was added to the sugar phosphate extract and incubated at 42°C for 4 h in a chemical fume hood. The reactions were then quenched by the addition of 50 µL 1M Tris, pH 7.0. Reactions were collected by centrifugation and transferred to autosampler vials for immediate HPLC analysis. Reactions were run on a Shimadzu LC-20Ai inert HPLC system. Buffer A consisted of 20 mM ammonium acetate, pH 7.0, and Buffer B consisted of acetonitrile with 0.1% formic acid. We performed isocratic elution with 70% B for 30 min at 350 µL/min, maintaining a column temperature of 25°C, using a 20 µL injection volume. The stationary phase consisted of a Shim-Pack Velox HILIC column with 2.7 µM particle size, 4.6 ID × 150 mm length. AMPy-Ribose 5-phosphate conjugate is found to fluoresce at 420_nm_ with an excitation wavelength of 350_nm_. Ribose-5-phosphate concentrations were calculated using the peak intensity values after correcting for baseline shift in the Shimadzu analysis software.

#### Gas chromatography–mass spectrometry

Cells were grown in culture flasks in 150 mL of medium LB to OD_600nm_ ≈ 0.5. Nalidixic acid was added at a concentration of 25 mg/mL and incubated for 30 min. Intracellular metabolite extraction with cold methanol-water solution (50%, vol/vol) was carried out according to the method (41). Aliquots (750 µL) were vacuum-dried. Extraction of metabolites and their derivatization with *O*-methylhydroxylamine (Sigma) and *N*-trimethylsilyl-*N*-methyl trifluoroacetamide (MSTFA) (Supelco, Bellefonte, PA, USA) were performed in accordance with the general protocol of Fiehn lab (42). As an internal standard, nor-valine (Sigma) was added during the extraction stage. Briefly, a chilled mixture of acetonitrile/2-propanol/water (3:2:2) containing nor-valine was added to each sample with subsequent vortexing (10 s), incubation in ultrasonic bath (5 min), and shaking at 4°C (1,000 rpm, 5 min). After removal of debris by centrifugation (14,000 *g*, 2 min, 4°C), the clarified extracts were dried on a vacuum concentrator, dissolved in a mixture of acetonitrile/water (1:1), and clarified by centrifugation again, and the extracts were vacuum-dried in new tubes. Derivatization of metabolites was carried out by incubation with *O*-methylhydroxylamine solution in pyridine (20 mg/mL) for 90 min at 30°C and then with MSTFA reagent for 30 min at 37°C at vigorous shaking. The final samples were transferred to glass vials with 200 µL inserts and subjected to GC-MS analysis.

Quantification of metabolites was performed by targeted GC–MS on a Chromatec Crystal-5000 gas chromatograph equipped with a 30 m CR-Xi-5ms column (0.25 mm, 0.25 µm) (Chromatec, Yoshkar-Ola, Russia), coupled to a monoquadrupole Chromatec mass-spectrometry detector (Chromatec) operating under electron impact ionization at 20 eV. The samples (1 µL) were injected at 250°C in splitless mode into a 1 mL/min helium flow. The oven temperature was maintained at 60°C for 1 min, increased to 310°C at 10 °C/min, and kept for an additional 10 min at this temperature. The transfer line and ion source temperature were held at 270°C and 200°C, respectively. After 8 min of solvent delay, the MS data were recorded in full scan mode in the range of 50–600 *m*/*z*. Ribose-5-phosphate was quantified in SIM mode by detecting a characteristic ion with certain retention time: 22.2 min and 299 *m*/*z* for ribose-5-phosphate. These data were obtained by analysing their standard (D-ribose-5-phosphate, Sigma) prepared using the same derivatization protocol, analytical parameters, and instruments. Raw spectra were analyzed using Chromatec Analytic software (version 3.0.0.2). The concentration of each compound was calculated from their peak intensities using a calibration curve obtained under the same experimental settings. The values were corrected to the signal of nor-valine used as an internal standard and then normalized to cell number.

### Statistical analysis

The data are shown as mean ± standard deviation measures from triplicate values obtained from 3–4 independent experiments. The statistical difference between experimental groups was analyzed by one-way ANOVA with Tukey correction for multiple comparisons. Probability values (*P*) less than 0.05 were considered significant. Statistical analysis was performed using the GraphPad Prism 9.1.2 software (GraphPad Software Inc., San Diego, CA, USA).

## Data Availability

All study data are included in the main text and supplemental material.

## SUPPLEMENTAL MATERIAL

Supplemental material mbio.00654-25-s0001.pdf Fig. S1 to S9; Table S1 to S3.

ASM does not own the copyrights to Supplemental Material that may be linked to, or accessed through, an article. The authors have granted ASM a non-exclusive, world-wide license to publish the Supplemental Material files. Please contact the corresponding author directly for reuse.
