# Supplementary material for: Ribose-5-phosphate metabolism protects E. coli from antibiotic lethality
Source: mBio. 2025 Jul 2;16(8):e00654-25. doi: 10.1128/mbio.00654-25 (PMC12345141; doi:10.1128/mbio.00654-25)
Supplement: Supplemental material — Fig. S1 to S9; Table S1 to S3. [file mbio.00654-25-s0001.pdf]

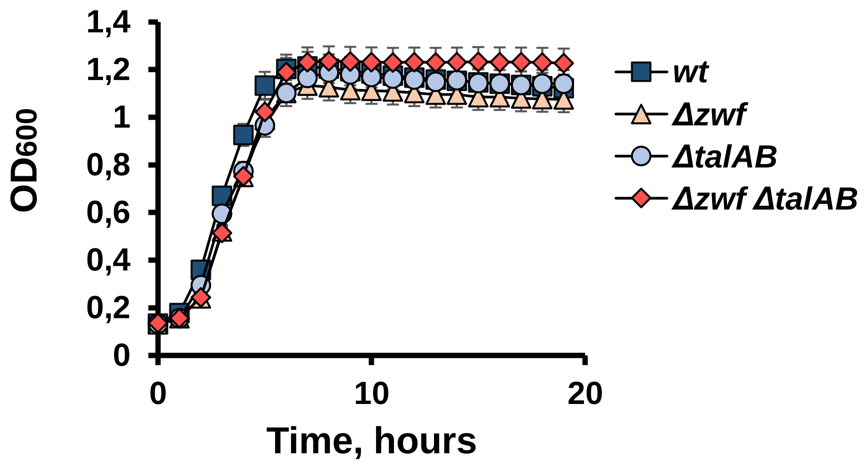

Fig. S1. Representative optical density (OD_{600}) growth curves of wt, *Δzwf, ΔtalAB, Δzwf ΔtalAB* strains grown in complete LB medium. Overnight cultures were diluted with fresh LB 1:100 and grown in LB-medium for 20 h. Cells were grown in triplicate at 37°C with aeration using a Bioscreen C automated growth analysis system. The curves represent the averaged values from three parallel experiments.

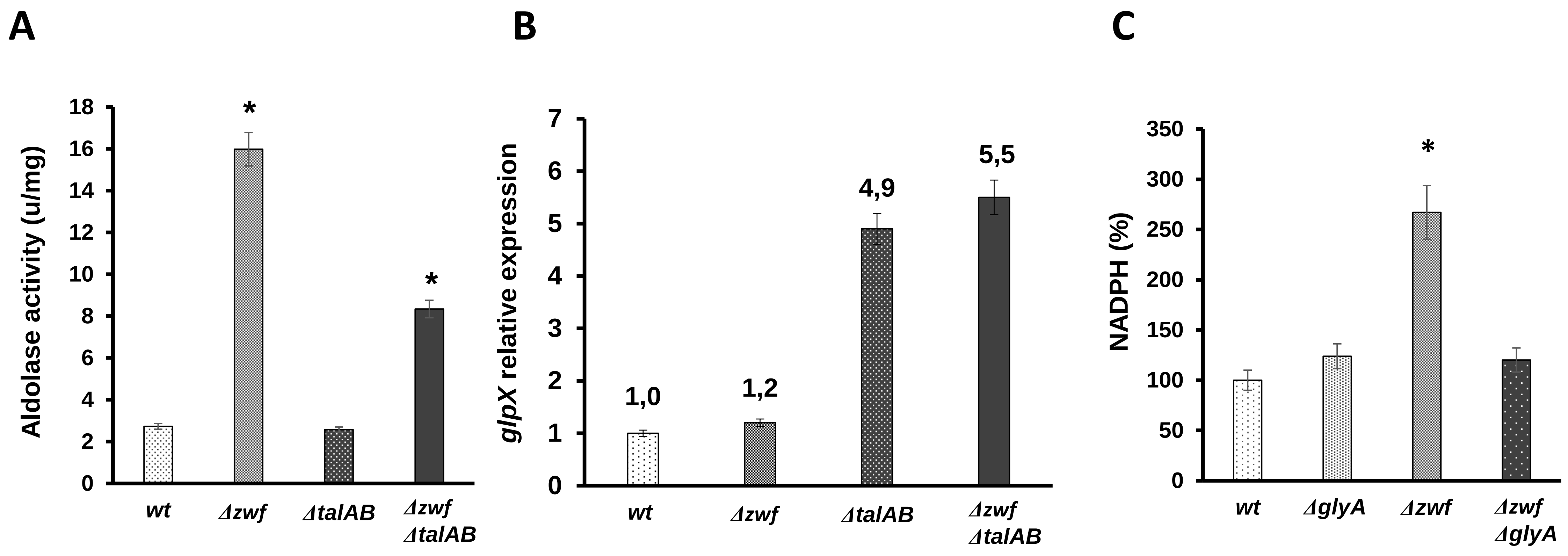

Fig. S2. Glycolitic enzymes aldolase A, phosphatase GlpX and serine hydroxymethyltransferase are involved in aPPP. (A) Activity of aldolase encoded by the *fbaA* gene. Mean values ± SD from at least three independent experiments are shown. * – $p < 0.05$, compared to the wild-type cells. (B) The relative expression of the *glpX* gene in exponentially grown WT and mutant cells was measured by qRT-PCR. The relative expression (y-axis) represents the fold change of each mRNA level compared with that of the wt cells. Values are means ± SD from four experiments. (C) Intracellular content of NADPH in the Δ*zwf* mutant upon inactivation of the *glyA* gene. Mean values ± SD from at least three independent experiments are shown. * – $p < 0.05$, compared to the wild-type cells.

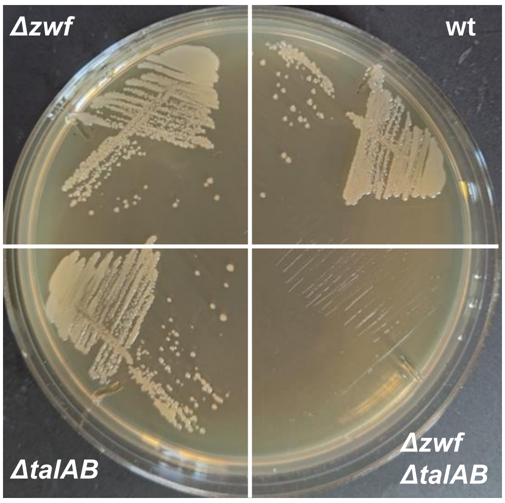

Fig S3. Appearance of KanR recombinants in transduction experiments. P1 grown on Keio strain JW3896 *glpX*::kan with recipient: WT, *Δzwf*, *ΔtalAB* and *Δzwf ΔtalAB*. After transduction, the cells were plated on LB medium with Kan and incubated at 37°C overnight. It was not possible to obtain viable colonies for the triple mutant *Δzwf ΔtalAB*. The presence of the *glpX* gene deletion was confirmed by PCR.

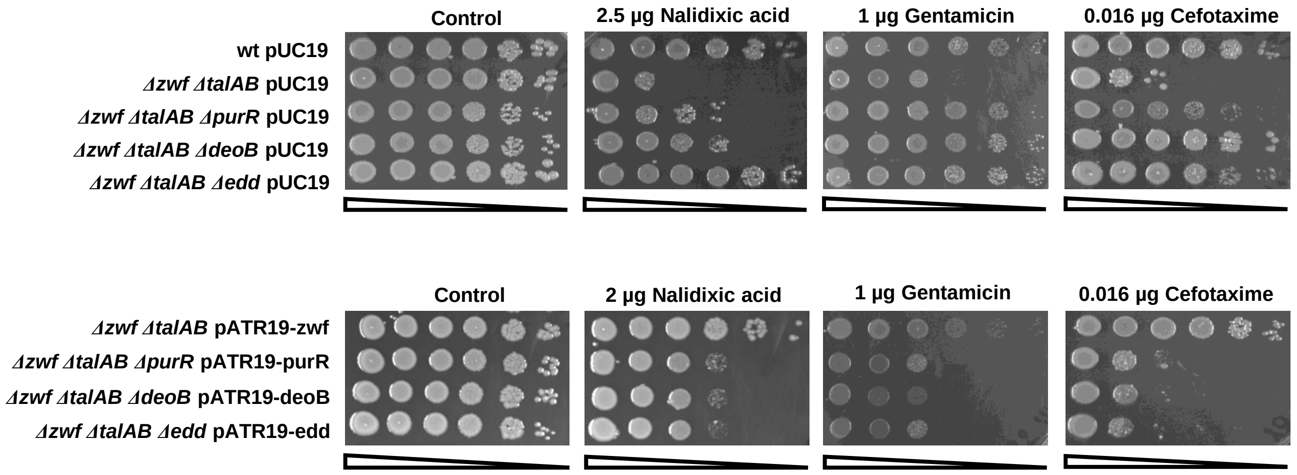

Fig. S4. Genetic complementation of deletions *zwf, purR, deoB edd*. Representative efficiencies of colony formation of strains, caring vectors with wild-type alleles of genes *zwf*, *purR*, *deoB* and *edd*. To determine representative efficiencies of colony formation, cells were grown to OD_{600} ~ 0.4, and serial 10 fold dilutions were spotted on LB agar plates containing the indicated concentrations of nalidixic acid, gentamicin or cefotaxime and incubated at 37 °C for 24 h.

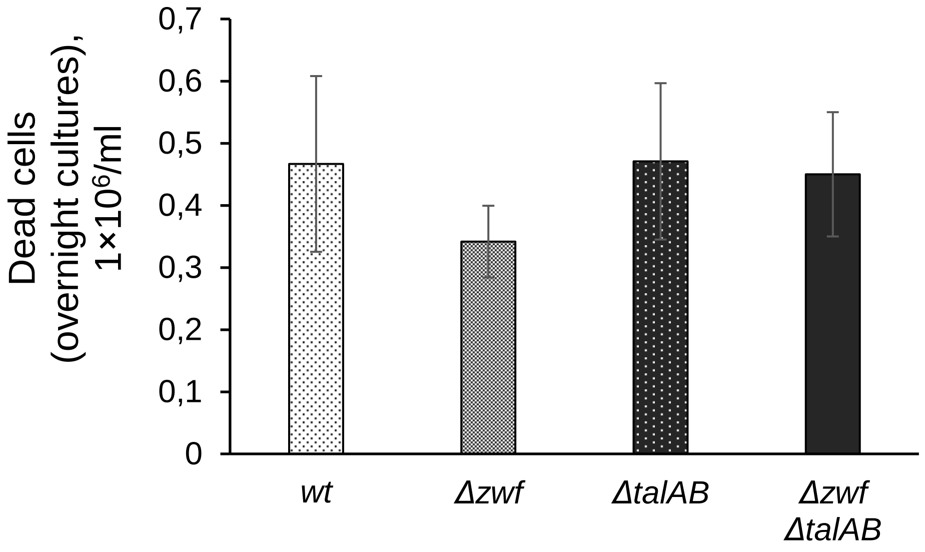

Fig S5. The number of dead cells in the cell population of overnight cultures was determined by flow cytometry using propidium iodide. Cells were grown in an LB medium to an optical density of 0.4 and then washed twice with phosphate-buffered saline (1xPBS), centrifuged, the supernatant was removed, and cells were resuspended in 100 μL of PBS. Cells were incubated in the presence of propidium iodide at a concentration of 10 μg/mL within 1 minute before the start of the analysis.

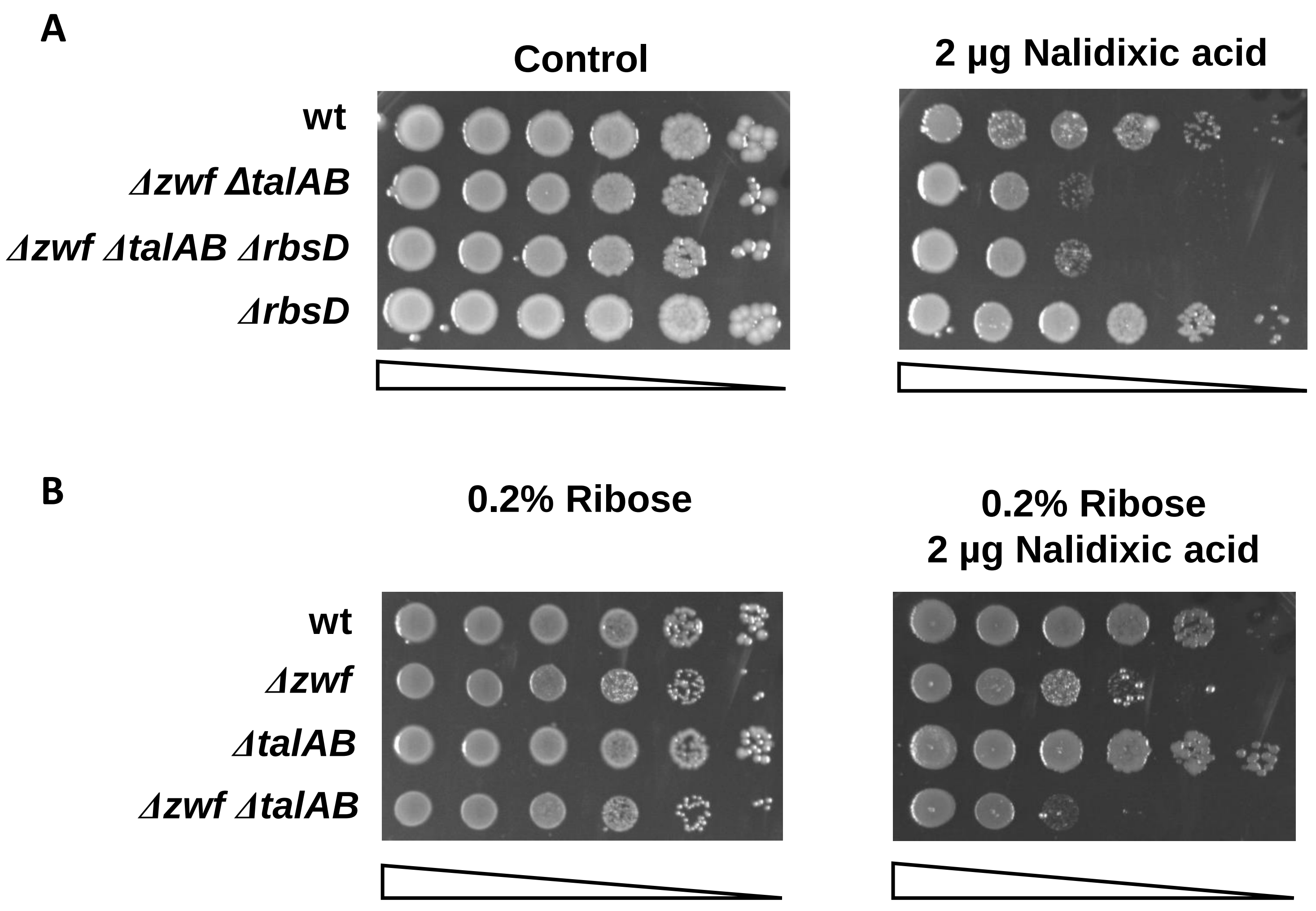

Fig S6. Ribose present in LB medium does not affect the sensitivity of aPPP mutants to antibiotics. (A) Representative efficiencies of colony formation on LB medium triple mutant *Δzwf ΔtalAB* with inactivated ribose transport (*ΔrbsD*) in the presence of nalidicxic acid. **(B)** Representative efficiencies of colony formation on LB medium with 0.4% ribose by mutants *Δzwf* , *ΔtalAB* and *Δzwf ΔtalAB* in the presence of nalidixic acid.

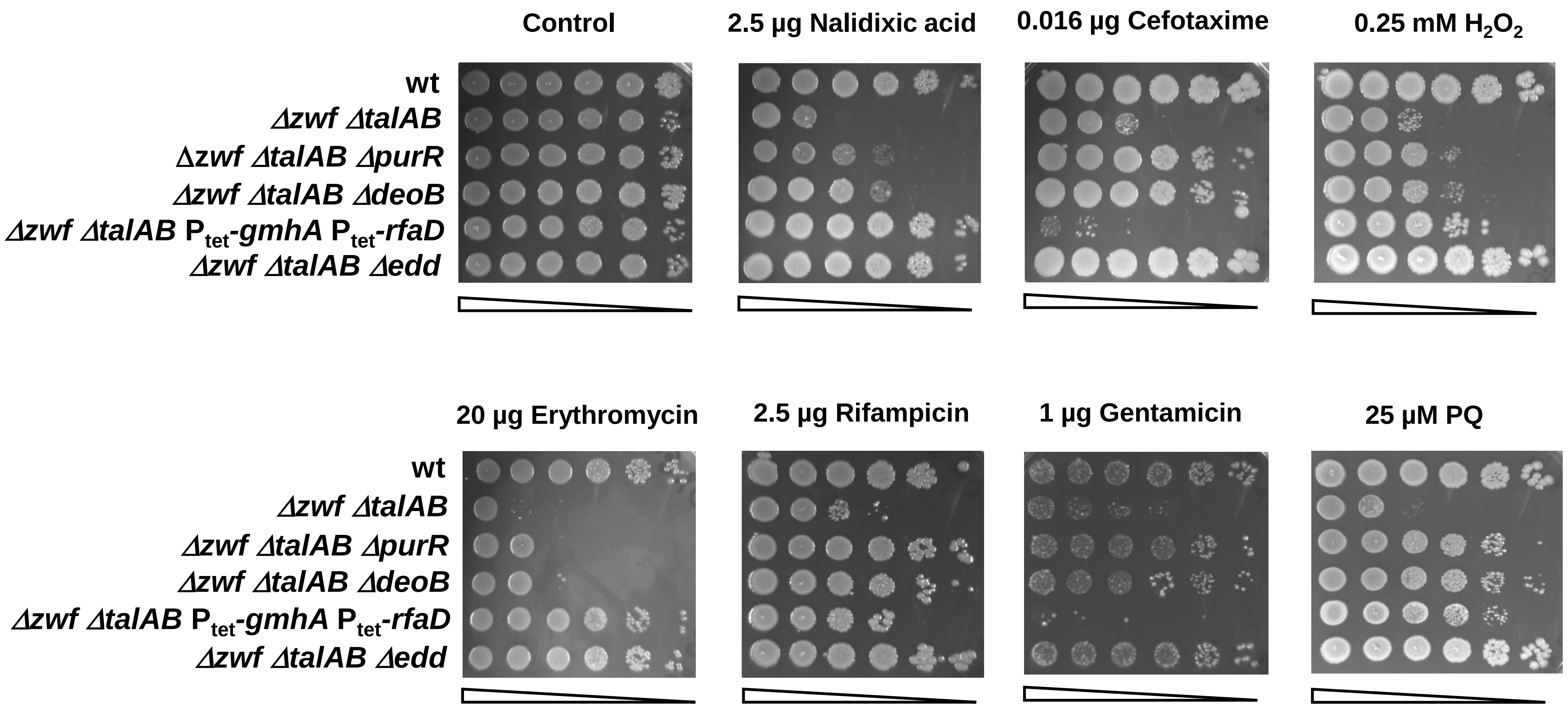

Fig. S7. The survivability of Δ*zwf* Δ*talAB* cells is rescued by inactivation of *purR*, *deoB*, or *edd* gene or by *gmhA* and *rfaD* overexpression. To determine representative efficiencies of colony formation, cells were grown to OD_{600} ~ 0.4, and serial 10-fold dilutions were spotted on LB agar plates containing the indicated concentrations of antibiotics or H_2O_2 and paraquat and incubated at 37 °C for 24 h.

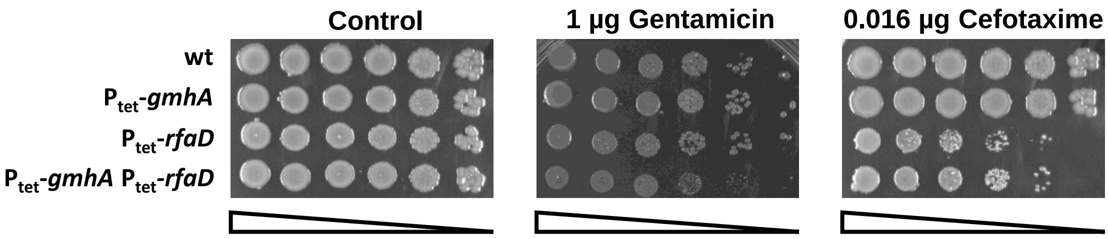

Fig. S8. The influence of single mutations P_{tet}-*gmhA* and P_{tet}-*rfaD* and their combination on the sensitivity of strain MG1655 to gentamicin and cefotaxime. Representative efficiencies of colony formation on LB medium in the presence of gentamicin and cefotaxime.

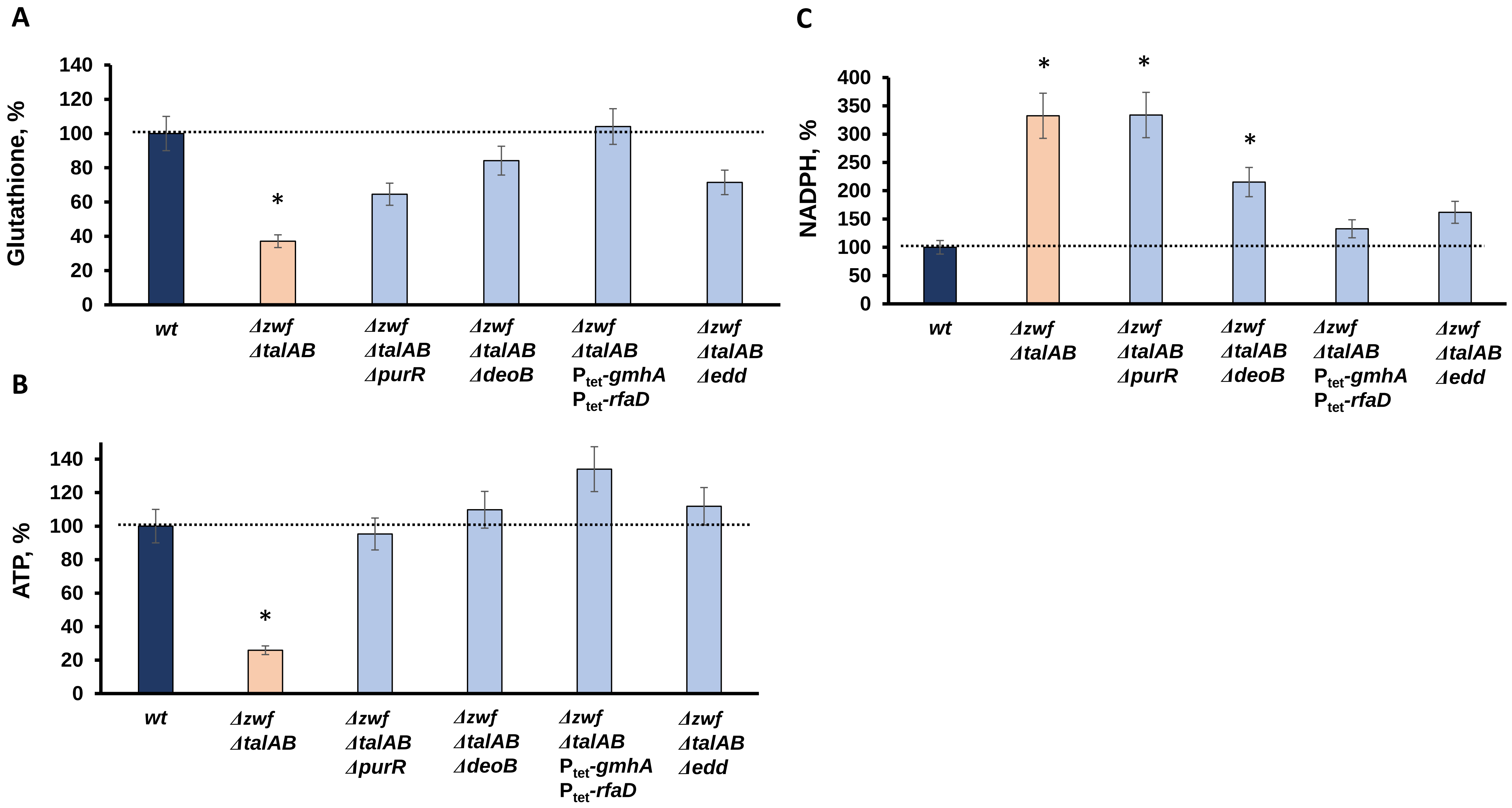

Fig. S9. Intracellular content of glutathione (A), ATP (B), and NADPH (C) in the Δ*zwf* Δ*talAB* mutants against the background of *purR*, *deoB*, *edd* and P_{tet}-*gmhA* P_{tet}-*rfaD* mutations. Mean values ± SD from at least three independent experiments are shown. * – $p < 0.05$, compared to the wild-type cells.

Table S1. MICs for strains used in this study.

**Strain**	**Nalidixic acid (μg/ml)**	**Gentamicin (μg/ml)**	**Cefotaxime (μg/ml)**	**Erythromycin (μg/ml)**	**Rifampicin (μg/ml)**	**H_2O_2 (mM)**	**Paraquat (μM)**
MG1655	8.75 ± 0.25	1.25 ± 0.05	0.08 ± 0.005	95 ± 2.5	13 ± 0.5	1 ± 0.1	250 ± 5
Δ*zwf*	9 ± 0.25	1.25 ± 0.05	0.08 ± 0.005	95 ± 2.5	12 ± 0.5	0.5 ± 0.05	130 ± 5
Δ*talAB*	9 ± 0.25	1.30 ± 0.05	0.075 ± 0.005	95 ± 2.5	12 ± 0.5	1 ± 0.1	250 ± 5
Δ*zwf* Δ*talAB*	9 ± 0.25	1.25 ± 0.05	0.08 ± 0.005	95 ± 2.5	12 ± 0.5	0.35 ± 0.05	100 ± 5
Δ*zwf* Δ*talAB* Δ*purR*	8.75 ± 0.25	1.4 ± 0.05	0.08 ± 0.005	92.5 ± 2.5	12 ± 0.5	0.75 ± 0.05	180 ± 5
Δ*zwf* Δ*talAB* Δ*deoB*	9 ± 0.25	1.3 ± 0.05	0.08 ± 0.005	92.5 ± 2.5	12 ± 0.5	0.75 ± 0.05	200 ± 5
Δ*zwf* Δ*talAB* P_{tet}-*gmhA* P_{tet}-*rfaD*	8.75 ± 0.25	1.4 ± 0.05	0.07 ± 0.005	95 ± 2.5	12 ± 0.5	0.75 ± 0.05	190 ± 5
Δ*zwf* Δ*talAB* Δ*edd*	8.5 ± 0.25	1.4 ± 0.05	0.075 ± 0.005	95 ± 2.5	12 ± 0.5	1 ± 0.1	250 ± 5

Table S2. Bacterial strains and plasmids.

***E.coli* strain**	**Genotype**	**Source**
MG1655	F^- wild type *E.coli*	Laboratory collection
BW25113	F^- Δ(*araD-araB*)*567*, *lacZ4787*(::rrnB-3), λ^-, rph-1, Δ(*rhaD-rhaB*)*568*, *hsdR514* with pKD46-(Ts)	Ref. 32
JW3896	As BW25113 plus *ΔglpX*::kan	Keio collection (30)
TS011	As MG1655 plus Δ*zwf*	This work
TS013	As MG1655 plus Δ*talAB*	This work
TS015	As TS011 plus Δ*talAB*	This work
TS021	As TS015 plus Δ*purR*	This work
TS025	As TS015 plus Δ*deoB*	This work
TS028	As TS015 plus P_{tet}-*gmhA* P_{tet}-*rfaD*	This work
TS031	As TS015 plus Δ*gnd*	This work
TS032	As TS015 plus Δ*edd*	This work
TS033	As MG1655 plus Δ*glpX*	This work
TS034	TS011 plus Δ*glpX*	This work
TS035	TS013 plus Δ*glpX*	This work
TS036	TS015 plus Δ*glpX*	This work
TS037	As MG1655 plus Δ*gshA* Δ*gshB*	This work
TS038	As TS015 plus pATR-zwf	This work
TS039	As TS021 plus pATR-purR	This work
TS040	As TS025 plus pATR-deoB	This work
TS041	As TS032 plus pATR-edd	This work
Plasmid		
pCP20	*FLP* $^+$, λ cI857 $^+$, λ p_R Rep^{ts}, Amp^R, Cm^R	Ref. 32
pUC19	Amp^R, lac promoter	Laboratory collection
pATR-zwf	pUC19 carrying *zwf*	This work
pATR-purR	pUC19 carrying *purR*	This work
pATR-deoB	pUC19 carrying *deoB*	This work
pATR-edd	pUC19 carrying *edd*	This work
pDEW201	Amp^R, promoterless *P. lumvtescens luxCDABE* operon	Ref. 37
pSoxS'::*lux*	As pDW201 plus *PsoxS*':: *luxCDABE*	Ref. 35

Table S3. Primers used in this study.

Primer	Sequence
GmhA1	5′-gcacttcaggtcaaaaagtcctggtcatagcacctgcgctcaagttagtataaaaaagct-3′
GmhA2	5′-ttcgttcag-ttcgttacgaataagatcctggtacatggtacctttctcctctttaatga-3′
GmhA3	5′-aacaaagctcacattgttgct-3′
GmhA4	5′-gcgctgaatggcgtgaatatt-3′
RfaD1	5′- ttcacatgcaaaaccaacatccgccatgaaggactacgctcaagttagtataaaaaagct-3’
RfaD2	5′-gccgat-aaagcccgcgccgccggtaacgatgatcatggtacctttctcctctttaatga-3′
RfaD3	5′-atcggaatattgatactaaagc-3′
RfaD4	5′-aatatcggtgatgcctttatc-3′
Zwf7	5’–agaggatccacagttttcgcaagctcgtaa–3’
Zwf8	5’–agtgaattcttactcaaactcattccagga–3’
PurR7	5’-agaggatccccacacaaaaagtgatattacg–3’
PurR8	5’-agtgaattcttaacgacgatagtcgcgag–3’
DeoB7	5’-agaggatccatgaaacgtgcatttattatggt–3’
DeoB8	5 –agtgaattctcagaacatggctttgccatat–3’
Edd7	5’–agaggatcctctgcgcttatcctttatggt–3’
Edd8	5’–agtgaattcttaaaaagtgatacaggttgcg–3’
